# Expression Regulation and Physiological Role of Transcription Factor FOXO3a During Ovarian Follicular Development

**DOI:** 10.3389/fphys.2020.595086

**Published:** 2020-11-05

**Authors:** Hong Zhang, Fengping Lin, Jiuhua Zhao, Zhengchao Wang

**Affiliations:** ^1^Provincial Key Laboratory for Developmental Biology and Neurosciences, Provincial University Key Laboratory of Sport and Health Science, Key Laboratory of Optoelectronic Science and Technology for Medicine of Ministry of Education, College of Life Sciences, Fujian Normal University, Fuzhou, China; ^2^West Anhui Health Vocational College, Lu’an, China

**Keywords:** transcription factor FOXO3a, cell cycle, apoptosis, oxidative stress, energy metabolism, follicular development

## Abstract

In mammals, developing ovarian follicles transform from primordial follicles to primary follicles, secondary follicles, and mature follicles, accompanied by changes in follicular secretory functions. FoxO3a is a member of the forkhead transcription factor family (FoxO), which plays an important role in the cell cycle, DNA damage repair, apoptosis, oxidative stress, and energy metabolism. Recent studies have shown that FOXO3a is involved in the physiological regulation of follicular development and pathological progression of related ovarian diseases, which will provide useful concepts and strategies for retarding ovarian aging, prolonging the ovarian life span, and treating ovarian diseases. Therefore, the regulation of FOXO3a expression, as well as the physiological contribution during ovarian follicular development are detailed in this paper, presenting an important reference for the further study of ovarian biology.

## Introduction

Follicular development is a complex reproduction-related physiological process characterized by cell proliferation, differentiation, and apoptosis. Typically, based on morphology and function, follicular development can be artificially divided into different stages, including primordial follicles, primary follicles, secondary follicles, and mature follicles (Wei et al., 2012, 2019; Huang et al., 2016; Wu et al., 2019). Notably, various diseases could be induced by follicular dysplasia, including premature ovarian failure, polycystic follicular syndrome, and infertility (Yang et al., 2010; Thanatsis et al., 2019). Forkhead box (Fox) proteins are highly conserved transcription factors structurally, currently attracting a great deal of attention. Among them, FOXO3a is an important member, and its discovery originates from its homologous protein DAF-16, which is also a well-studied transcription factor (Liu et al., 2018). Ogg et al. (1997) revealed that the FOXO3a homologous protein, DAF-16, is negatively regulated by the insulin signaling pathway. Furthermore, it participates in the regulation of the cell cycle and life expectancy, which is closely related to the lifespan, metabolism, and reproduction of worms (Ogg et al., 1997). Thus, FOXO3a may be closely related to the development, metabolism, and other functions of organisms. Recent studies have shown that FOXO3a is involved in follicular development, thus presenting a valuable target for the study of follicular development, and displays important theoretical and practical significance for better understanding the mammalian reproductive mechanism.

## The Development of Ovarian Follicles

In mammals, the primordial follicle is the basic female reproductive unit and the only form of ovarian cell reserve (Wei et al., 2012, 2019; Huang et al., 2016; Wu et al., 2019). Primitive follicular pools are formed during early life such as the late embryonic stage in humans and the fourth day after birth in rats (Tang et al., 2017). Furthermore, once primordial follicles are formed, their total number remains fixed and is no longer increased. Usually, after the formation of primordial follicles, there will be a continuous batch of developing primordial follicles, and then forming follicles at different developmental stages, finally becoming dominant follicles triggering ovulation to commence a new life journey (Zhang Z. et al., 2019). During follicular development, most follicles die a programmed death or degeneration, which is termed follicular apoptosis or atresia (Tang et al., 2017). There are two types of follicular atresia from the morphological standpoint, starting from oocytes or granulosa cells, respectively (Manabe et al., 2004; Shimizu et al., 2009).

## Transcription Factor FOXO3a

The first forkhead protein was discovered in *Drosophila melanogaster* by Weigel et al. (1989). To date, more than 100 family members have been verified, from FOXA to FOXS (Anderson et al., 1998; Lee and Dong, 2017; Murtaza et al., 2017). FOXO belongs to the “O” class of the FOX superfamily. In mammals, this group contains four members: FOXO1/FKHR/FOXO1a, FOXO3/FKHRL1/FOXO3a, FOXO4/AFX, and FOXO6 (Murtaza et al., 2017). All FoxO proteins share a highly conserved DNA-binding domain, presenting 110 amino acids folded into three α-helixes and two wing-like large loops. In addition, the structure includes a nuclear localization signal, a nuclear export signal motif, and a C-terminal transcriptional active region (Obsil and Obsilova, 2008). These proteins are ubiquitously expressed in various tissues throughout the body, except for FOXO6, which currently has been reported only in the adult brain tissue (Jacobs et al., 2003). Notably, the Human Protein Atlas^1^ indicates that the expression of FOXO3 in human ovarian stromal and follicular cells is abundant.

## Expression Regulation of FOXO3a During Follicular Development

**FIGURE 1:**
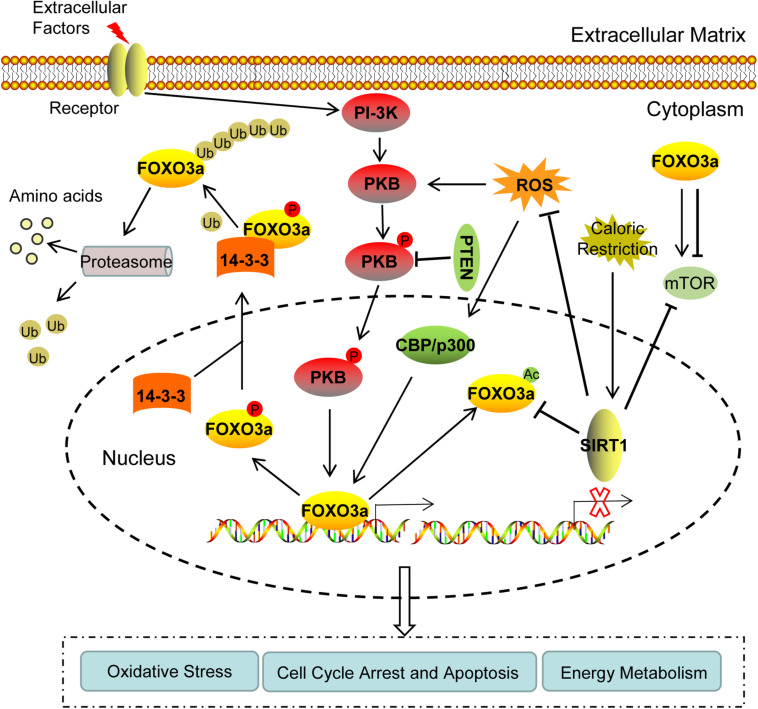
The regulation of FOXO3a expression and its physiological role of during follicular development. (1) FOXO3a can be phosphorylated by PKB, and then transported from the nucleus to the cytoplasm by 14-3-3 molecular chaperone, accompanied by the loss of transcriptional activity. PTEN can inhibit the inactivation of FOXO3a by dephosphorylating p-PKB. (2) In the cytoplasm, the polyubiquitination of FOXO3a results in its degradation by proteasomes. (3) Acetylase CBP, p300, and other nuclear proteins can acetylate FOXO3a protein. In the nucleus, SIRT1 may activate FOXO3a by deacetylating Ac-FOXO3a. (4) Under oxidative stress, the accumulated ROS can increase post-translational modifications of FOXO3a, whereas SIRT1 can downregulate ROS. (5) FOXO3a is involved in autophagy by regulating mTOR. Caloric restriction may activate SIRT1 signaling and suppress mTOR. Collectively, the regulation of FOXO3a can mediate cell cycle arrest and apoptosis by inducing the transcription of downstream target genes, as well as by participating in oxidative stress and energy metabolism through communication with PKB, SIRT1, ROS, and mTOR, thereby affecting the activation of primordial follicles, oocyte and granulosa cell apoptosis, and regulating the growth and development of follicles. PKB, protein kinase B; PTEN, phosphatase and tensin homolog deleted on chromosome ten; SIRT1, sirtuin 1; ROS, reactive oxygen species; mTOR, mammalian target of rapamycin.

During recent years, several studies have investigated the regulation of FOXO3a expression. The activity of FOXO3a can be improved at multiple levels, in which post-translational modification is the main approach, including phosphorylation, acetylation, and ubiquitination (Figure 1).

### Phosphorylation and Dephosphorylation

FOXO3a can be phosphorylated by kinases such as protein kinase B (PKB), extracellular-regulated kinase, serum and glucocorticoid-induced kinase, and inhibitor kappa B kinase β (Brunet et al., 2001). The transcriptional regulation of FOXO3a is closely related to phosphoinositide-3 kinase (PI-3K)/protein kinase B (PKB) signaling, which was first proposed by Brunet et al. (1999). In mammals, FOXO3a can be phosphorylated by PKB in the nucleus and then transported from the nucleus to the cytoplasm, utilizing the 14-3-3 molecular chaperone after the activation of insulin signaling. FOXO3a is translocated into the cytoplasm and could bind with the polyubiquitination system, to be subsequently degraded by proteasomes (Plas and Thompson, 2003; Wang et al., 2015), which accompanies the transcriptional activity loss of FOXO3a, which no longer plays a regulatory role during cell development and metabolism (Datta et al., 1999; Brunet et al., 2002; Plas and Thompson, 2003, 2005). Therefore, the localization of FOXO3a in the cytoplasm not only inactivates its function but is also extremely crucial for the degradation of FOXO3a.

Phosphatase and tensin homolog deleted on chromosome ten (PTEN) is a key negative regulator for PI3K/PKB signaling, which can improve the suppression of FOXO3a through dephosphorylation (Ding et al., 2010; Jang et al., 2016; Li J. et al., 2020). Additionally, endogenous PKB and FOXO3a can form a complex. Furthermore, FOXO3a can negatively regulate PKB and its downstream molecules (Takaishi et al., 1999; Lin et al., 2001; Junger et al., 2003; Puig et al., 2003). Simultaneously, elevated 14-3-3 can increase FOXO3a expression and phosphorylation, maintaining the phosphorylated FOXO3a protein stability (Cahill et al., 2001).

Reddy et al. (2005) reported the activation of PKB and suppression of FOXO3a in mouse and rat oocytes using stem cell factor (SCF) during follicular development. Meng et al. (2007) showed the stage/cell-specific expression patterns of FOXO3a and PKB, suggesting that these proteins might play potential roles in the follicular development of the mini-pig. Furthermore, consistent results were observed in fetal and neonatal pig ovaries (Ding et al., 2010). These results indicate the important role of PKB/FOXO3a and the impact of PKB regulation on FOXO3a phosphorylation during follicular development.

### Acetylation and Deacetylation

In addition to the regulation of PKB, FOXO3a is also mediated via acetylation and deacetylation (Kim et al., 2010; Xiang et al., 2012; Zhang et al., 2013; Wang et al., 2014; Zhou et al., 2014; Liu et al., 2015; Long et al., 2019). CREB binding protein, p300, and other nuclear proteins can acetylate lysine on the DNA-binding region of FOXO3a protein, resulting in reduced FOXO3a transcriptional activation (Watroba et al., 2012). Sirtuin 1 (SIRT1) is a NAD-dependent histone deacetylase (Long et al., 2019). Typically, SIRT1 in the nucleus may activate the transcriptional activity of FOXO3a, regulating cellular functions by deacetylating Ac-FOXO3a (Glauser and Schlegel, 2007). Gorczyca et al. (2019) demonstrated the presence of SIRT1 and SIRT6 in ovarian cells, and their involvement in the control of follicular atresia. Recent studies revealed that energy restriction can increase the expression of SIRT1 and activate SIRT1-related signaling pathways in adult mice (Xiang et al., 2012; Zhang et al., 2013; Liu et al., 2015; Long et al., 2019). Additionally, Kim et al. (2010) reported that the high expression of FOXO3a can upregulate SITR6 activity, whereas the inhibition of FOXO3a expression could downregulate SIRT6 activity. Simultaneously, the downregulation of FOXO3a can prevent the effect of SIRT6 on energy limitation, as well as on SIRT1 (Kim et al., 2010).

## Physiological Roles of FOXO3a During Follicular Development

Currently, numerous studies have presented that FOXO3a is associated with follicular development (Brenkman and Burgering, 2003; Adhikari and Liu, 2009; Monniaux et al., 2016). Experimental studies have reported that female FOXO3a knockout mice exhibit global follicular activation at an early stage of follicular growth, leading to oocyte death, early depletion of functional ovarian follicles, and secondary infertility (Castrillon et al., 2003). Conversely, FOXO3 overexpression can delay the development of primordial follicles, increase the follicular reserve, and ovarian reproductive capacity in mice. Compared with wild-type littermates, increased follicle numbers and decreased gonadotropin levels were documented in aging FOXO3-transgenic mice (Pelosi et al., 2013). Thus, FOXO3a may play an important role in maintaining the pool number of primordial follicles and the physiological functions of the ovarian reserve, as well as female fertility. Furthermore, some researchers have reported that the FOXO3 protein regulates follicle growth and atresia by promoting apoptosis of granulosa cells and oocytes in mammalian ovaries (Liu et al., 2009; Matsuda et al., 2011).

Although the function of FOXO3a in ovarian follicle development has been relatively known, its mechanism remains unclear. It is generally accepted that FOXO3a is widely involved in the cell cycle, DNA damage repair, apoptosis, oxidative stress, and metabolism. Hence, we presented evidence postulating that the role of FOXO3a in follicular development is related to these processes (Figure 1).

### Cell Cycle Arrest and Apoptosis

FOXO3a activity impacts the expression of downstream target genes, resulting in cell cycle and apoptotic disturbances (Medema et al., 2000). FoxO3a can increase the expression of the cyclin-dependent kinase inhibitor protein, p27kip, and decrease the expression of cyclin D in the nucleus, maintaining cells in a stationary phase and inhibiting follicular development (Schmidt et al., 2002). Liu et al. (2009) suggested that FOXO3a is involved in oocyte apoptosis in the neonatal rat ovary, and the SCF-PI3K/PKB-FOXO3a signaling pathway mediates primordial follicle formation and oocyte apoptosis by regulating the expression of p27kip1 and proapoptotic factors such as Bim, Bad, and Bax. Moreover, research on chicken primary ovarian granulosa cells indicated that in the absence of FOXO3, mRNA levels of proapoptotic factors BNIP3 and BCL2L11 decreased, along with poly [ADP-ribose] polymerase 1 (PARP-1) and cleaved caspase3 protein levels. After treatment with a recombinant FOXO3 protein, mRNA levels of BNIP3 and BCL2L11, as well as protein levels of PARP-1 and caspase3, were reportedly increased (Cui et al., 2019). Experiments in human ovarian granulosa-like tumor cells (KGN) have shown that expression of the proapoptotic factors FASLG and BCL2L11 is upregulated and cell death is induced by transfection of FOXO3 expression vectors (Matsuda et al., 2011). Collectively, these studies have consistently demonstrated that FOXO3 is expressed in reproductive tissues, including ovarian oocytes and granulosa cells, and promotes apoptosis.

### Oxidative Stress

Reportedly, accumulated evidence suggests that oxidative stress is associated with disrupted follicular development, which may result in increased follicular atresia (Yan et al., 2020). Under oxidative stress, accumulated reactive oxygen species (ROS) leads to post-translational modifications of FOXO3a, thereby regulating the activity and function of FOXO3a. Park et al. (2020) demonstrated that SIRT1 can downregulate ROS and form a complex with FOXO3a in cells, which can improve the ability of FOXO3a to induce cell cycle arrest and promote cell survival. Recent findings have indicated that resveratrol, a plant polyphenolic compound, can enhance SIRT1 and decrease ovarian oxidative stress as well as inhibit phosphorylation of p66Shc, both *in vivo* and *in vitro* (Wang et al., 2020). Thus, in terms of follicular development, there undoubtedly exists an interactive relationship between ROS, SIRT1, and FOXO3a. However, the specific mechanism needs to be elucidated.

### Energy Metabolism

Mammalian target of rapamycin (mTOR) is a major negative regulatory factor of autophagy (Choi et al., 2011). It has been previously reported that PKB-mediated activation of mTOR inhibits granulosa cell autophagy during follicular development (Choi et al., 2014). Growing evidence strongly indicates that FOXO3a is involved in autophagy. If abundant energy is available, the modification of FoxO3 inhibits its activity, thereby decreasing the transcription of autophagy genes and downregulating autophagy. However, PI3K-PKB-FOXO3 can promote autophagy by mediating mTOR inhibition (van der Vos et al., 2012). Long et al. (2019) reported that oocyte-specific SIRT1-overexpressing mice demonstrated an improved follicle reserve and a prolonged ovarian lifespan by continuously activating FOXO3a and suppressing mTOR. Furthermore, SIRT1 can facilitate primordial follicle recruitment through directly modulating PKB and mTOR transcription, independent of deacetylase activity (Zhang T. et al., 2019). High-fat diet-induced obesity may accelerate ovarian follicle development and the rate of follicle loss by activating mTOR and suppressing SIRT1 signaling. Caloric restriction may improve the adverse effects of high-fat diet-induced obesity on ovarian follicles (Xiang et al., 2012; Wang et al., 2014; Li et al., 2015; Liu et al., 2015). Thus, FOXO3a, mTOR, PKB, and SIRT1 may be implicated in autophagy and energy metabolism during follicular development.

## FOXO3a and Ovarian Disease

Reportedly, the deletion of FOXO3a, FOXL2, PTEN, and p27 leads to early exhaustion of the primordial follicle pool and premature ovarian insufficiency in transgenic mice (Thanatsis et al., 2019). Melatonin prevents cisplatin-induced primordial follicle loss by suppressing the PTEN/AKT/FOXO3a pathway in the mouse ovary (Jang et al., 2016). Li Y. et al. (2020) observed that oral oyster polypeptide can protect the ovaries from D-galactose-induced premature ovarian failure, mediated via anti-oxidative stress activity. Meanwhile, growing data demonstrate that excess androgen may be the primary cause of polycystic ovary syndrome (PCOS). During the early stage of mouse folliculogenesis, testosterone induces the redistribution of FOXO3a, suggesting the involvement of FOXO3a in the pathogenesis of PCOS (Yang et al., 2010).

It has been well established that ovarian cancer presents the highest mortality rate among gynecological malignancies. Reportedly, FOXO3a expression can be increased by *LSD1* knockdown, thereby inhibiting the proliferation and metastasis of ovarian cancer HO8910 cells (Liu et al., 2020). Kaplan-Meier survival analysis suggested that the low expression of FOXO3a was significantly related to poor prognosis in ovarian cancer patients (Fei et al., 2009). Recently, Xia et al. (2020) revealed that microRNA-506-3p inhibits proliferation and promotes apoptosis in ovarian cancer cells by targeting the AKT/FOXO3a signaling pathway. O’Neill et al. (2013) suggested that blocking the epidermal growth factor receptor (EGFR) results in PI3K-PKB inhibition and increases FOXO3a activation, which provides a new and valuable treatment strategy for breast cancer, prostate cancer, and ovarian cancer.

## Summary and Conclusion

Based on the studies investigating the regulation of FOXO3a expression, it is currently established that FOXO3a can enhance the transcriptional regulation of its target genes, thereby enhancing its physiological contribution during the cell cycle and apoptosis regulation, resistance to oxidative stress, and prolongation of life span in organisms (Figure 1). Furthermore, FOXO3a signaling can induce oocyte and granulosa cell apoptosis, inhibit the activation of primordial follicles, and regulate the growth and development of follicles. The activation of FOXO3a signaling can inhibit the developmental initiation of primordial follicles, maintain the initial state of primordial follicles, reduce the number of primordial follicles transformed into mature follicles, thus preserving the follicular reserves, delaying the depletion of follicles, and delaying the aging of ovaries. It is important to further explore the mechanism concerning the regulation of FOXO3a expression on its target genes, the physiological contribution of FOXO3a during ovarian follicular development, and its future clinical applications, further advancing the field of reproductive biology.

## Author Contributions

HZ, FL, and JZ wrote the manuscript and ZW revised it. All authors read and approved the final version of the manuscript for publication.

## Conflict of Interest

The authors declare that the research was conducted in the absence of any commercial or financial relationships that could be construed as a potential conflict of interest.

## Footnotes

1 http://www.proteinatlas.org
